# Feasibility study of skin dosimetry with TLD sheets for measuring the effect of 3D printed bolus in radiotherapy

**DOI:** 10.1002/acm2.70035

**Published:** 2025-02-18

**Authors:** Yuya Miyasaka, Mayumi Ichikawa, Takagi Akira, Yoshifumi Yamazawa, Hongbo Chai, Hikaru Souda, Miyu Ishizawa, Hiraku Sato, Takeo Iwai

**Affiliations:** ^1^ Department of Heavy Particle Medical Science Yamagata University Graduate School of Medical Science Yamagata Japan; ^2^ Department of Radiology Yamagata University Faculty of Medicine Yamagata Japan; ^3^ Division of Dentistry and Oral Surgery Yamagata University Faculty of Medicine Yamagata Japan; ^4^ Department of Radiology Yamagata University Hospital Yamagata Japan

**Keywords:** 3D printer, bolus, radiotherapy, surface dose, TLD sheet

## Abstract

The thermoluminescence dosimeter (TLD) sheet is a measurement device coated with manganese‐doped LiB_3_O_5_ in sheet form. The sheet is 0.2‐mm thick and flexible. Hence, it can fit and be installed on irregular surfaces. The current study aimed to evaluate the feasibility of measuring patient surface doses during radiation therapy using TLD sheets. Further, the surface doses when using a three‐dimensional (3D) printed bolus were compared. First, calibration of the between TLD sheet measurements and the irradiation doses was performed. Second, dose calculations and TLD sheet measurements were compared to evaluate the error between treatment planning system (TPS) dose calculations and surface dose measurements. Finally, the TLD sheet was fitted to the head phantom to measure the surface dose at the uneven areas under two conditions (with a commercial [CM] bolus and a 3D‐printed bolus). Based on the error from the dose calculation algorithm and the difference between the TLD sheet thickness and the dose grid size or evaluation structure volume, the differences between the TLD sheet measurements and the treatment plan doses were up to 68.6% for the X‐ray collapsed cone convolution (CCC) method and up to 8.4% for the electron beam Monte Carlo (MC) method. Based on the results of the surface dosimetry of the head phantom, the application of surface dosimetry, particularly skin dosimetry, even on uneven body surfaces, can be feasible.

## 1 INTRODUCTION

Radiation therapy is used to treat patients by delivering doses to the skin surface, such as the chest wall and scalp.^1^, ^2^ However, the dose delivered on the skin surface is reduced due to the buildup of X‐ray and electron beam. In such cases, the skin surface dose is increased by placing a bolus on the tumor surface. Skin surface irradiation can be more effective with the use of a bolus.^3^, ^4^ However, commercially available boluses are plate‐shaped. Hence, they are occasionally difficult to fit to irregular surface shapes such as the nose, ears, scalp, and extremities of the human body. To address this issue, a novel bolus has been developed using three‐dimensional (3D) printers in recent years. Using a 3D printer, individualized boluses can be created from data on the surface geometry of individual patients. Park and Yea created a 3D‐printed bolus for irradiation of the ear and reported a reduced air gap between the bolus and skin surface and improved target coverage.^5^ Kong et al. simulated the dose distribution of tumors in the nose, ear, and parotid gland with a 3D‐printed bolus of silica gel and hydrogel in place.^6^ Their study showed that the target coverage was good for intensity‐modulated radiotherapy (IMRT) treatment planning. Robar et al. created a 3D‐printed bolus for the chest wall.^7^ This 3D‐printed bolus had a lesser air gap and shorter setup time than the sheet bolus. In addition, several reports showing the usefulness of a 3D‐printed bolus have been published. Some of these studies have reported that the 3D‐printed bolus was effective.^8^, ^9^, ^10^ However, these reports only compared treatment plans and evaluated deep depth doses with thick measurement devices. The skin surface dose itself when using the 3D‐printed bolus should be evaluated. Nevertheless, there are no reports directly evaluating skin surface doses. The use of thermoluminescence dosimeter (TLD) sheets for skin surface dosimetry has been proposed. TLD sheets are coated with TLD elements in sheet form and are 0.2‐mm thick and extremely flexible. The TLD element is LiBO_3_, with an effective atomic number of 7.3, which is close to that of water (7.5). It can be evaluated as a tissue‐equivalent material in terms of interaction with radiation. Kato et al. evaluated the characteristics of the TLD sheet as a measurement device.^11^ Skin dose prediction is clinically important for adequate dose delivery to the target when providing radiation therapy to the skin and for predicting skin toxicity. In particular, radiation therapy of the head and chest involves irradiation near the skin, so highly accurate skin dose prediction or in vivo dosimetry is required. Even if dose calculations are used, accurate predictions are difficult because most dose calculation algorithms assume charged particle equilibrium. With this in mind, the TLD sheet can be a technological breakthrough to overcome these challenges because it can directly measure surface dose. The TLD sheet enables measurement of the nose and ears, which are difficult to fit with Gafchromic films that have been widely used for surface dosimetry. Therefore, the current study aimed to examine the usefulness of a 3D‐printed bolus by measuring skin surface doses using TLD sheets.

## 2 MATERIAL AND METHODS

### 2.1 Thermoluminescence dosimeter (TLD) sheet measurement

**FIGURE 1:**
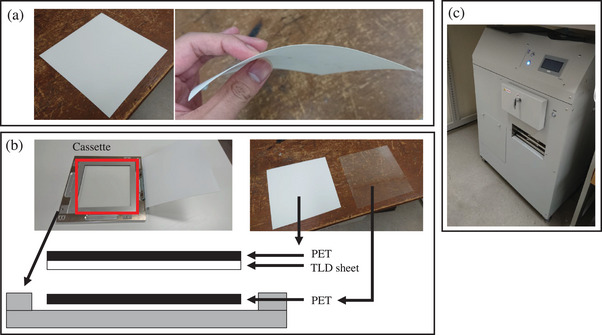
TLD sheet and measuring instrument. (a) shows a TLD sheet, (b) shows a cassette for inserting a measurement device, and (c) shows a TLD sheet measurement device. PET, polyethylene terephthalate; TLD, thermoluminescence dosimeter.

Similar to the product reported by Kato et al., the TLD sheet (Toyo Medic Co., Ltd., Tokyo, Japan) used in this study is a product in which the TLD element is coated with manganese‐doped LiB_3_O_5_ in sheet form.^11^ TLD sheets are flexible and can be easily fitted and installed on uneven surfaces (Figure 1). The irradiated TLD sheet was placed between the polyethylene terephthalate sheets in a cassette, as shown in Figure 1(b). Measurements were obtained by inserting the cassette into TLDR‐1 (Toyo Medic Co., Ltd., Tokyo, Japan) (Figure 1c). The cooling temperature of the charge‐coupled device camera was −20°C, the set temperature of measurement was 210°C, and the measurement time was 10 min. To reduce measurement error due to fading effects between irradiation and measurement, all measurements were performed 5 h after irradiation.

### 2.2 Association between TLD sheet measurements and irradiation doses

We performed a calibration on the TLD sheet measurements and irradiation doses. Since the TLD sheet measurements were assumed to be dependent on the irradiation beam energy, calibration was performed for each beam energy used in this study. This study measured each for the 4‐MV X‐ray therapy and 6‐MeV electron beam therapy. TLD sheets were cut into 5 × 5 cm pieces and placed in the tough water phantom (Kyoto Chemical, Kyoto, Japan) at a depth of 10 cm for the 4‐MV X‐ray beam and 2 cm for 6‐MeV electron beam. The irradiation system used was Elekta Synergy (Elekta AB, Stockholm, Sweden). Irradiation was performed with a gantry angle of 0°, a source‐to‐surface distance of 90 cm, and an irradiation field of 10 × 10 cm. That is, the distance from the source to the measurement point was 100 cm for the X‐ray beam and 92 cm for the electron beam. The irradiation system was adjusted to within 1.00 Gy ± 1% of the dose at the maximum dose point at 100 monitor unit (MU) in the monitor dosimeter. The MUs that should be irradiated were 20, 40, 60, 80, 100, 150, 200, 250, 300, 400, 500, and 700 MU. To obtain the association between irradiation MU and irradiated dose, the irradiated dose at the position of the TLD sheet was measured with the Farmer ionization chamber 30013 (PTW, Freiburg, Germany).

### 2.3 Surface dosimetry with the TLD sheets and treatment plan values

The treatment plan dose was compared, and the doses in surface dosimetry were measured using TLD sheets. The TLD sheets were placed in the center of the tough water phantom surface. We irradiated the phantoms with a gantry angle of 0°, a source‐to‐surface distance of 90 cm, and an irradiation field of 10 × 10 cm. Elekta Synergy was used as the treatment device, and the irradiation MU was set at 200 MU. Irradiation was performed with the 4‐MV X‐ray and 6‐MeV electron beam. Six irradiations and measurements were performed under each condition. The treatment plan was created using a treatment planning system (TPS) (RayStation10A, RaySearch Laboratories, Stockholm, Sweden) under the same conditions for irradiation. The computed tomography (CT) images used for dose calculation were those taken with Aquilion LB (Canon Medical Systems, Otawara, Japan) in the same geometry as the measurement. The dose calculation algorithms were collapsed cone convolution (CCC) for X‐ray and Monte Carlo (MC) for electron beam. The number of particles in the MC dose calculation was 10 000 per cm^2^. The grid size in the dose calculation was 1 mm. The dose calculation grid was set up to provide a quantitative and detailed understanding of the characteristics of the TLD sheet. The TLD sheet measurements were compared to the average dose values for the region of interest (ROI) in the TLD sheet calculated in the treatment plan. The dose values measured on the TLD sheet were compared with the treatment plan dose values obtained from the table showing the association between the TLD sheet measurements and dose values obtained (Section 2.2).

### 2.4 Development of the three‐dimensional (3D)‐printed bolus

**FIGURE 2:**
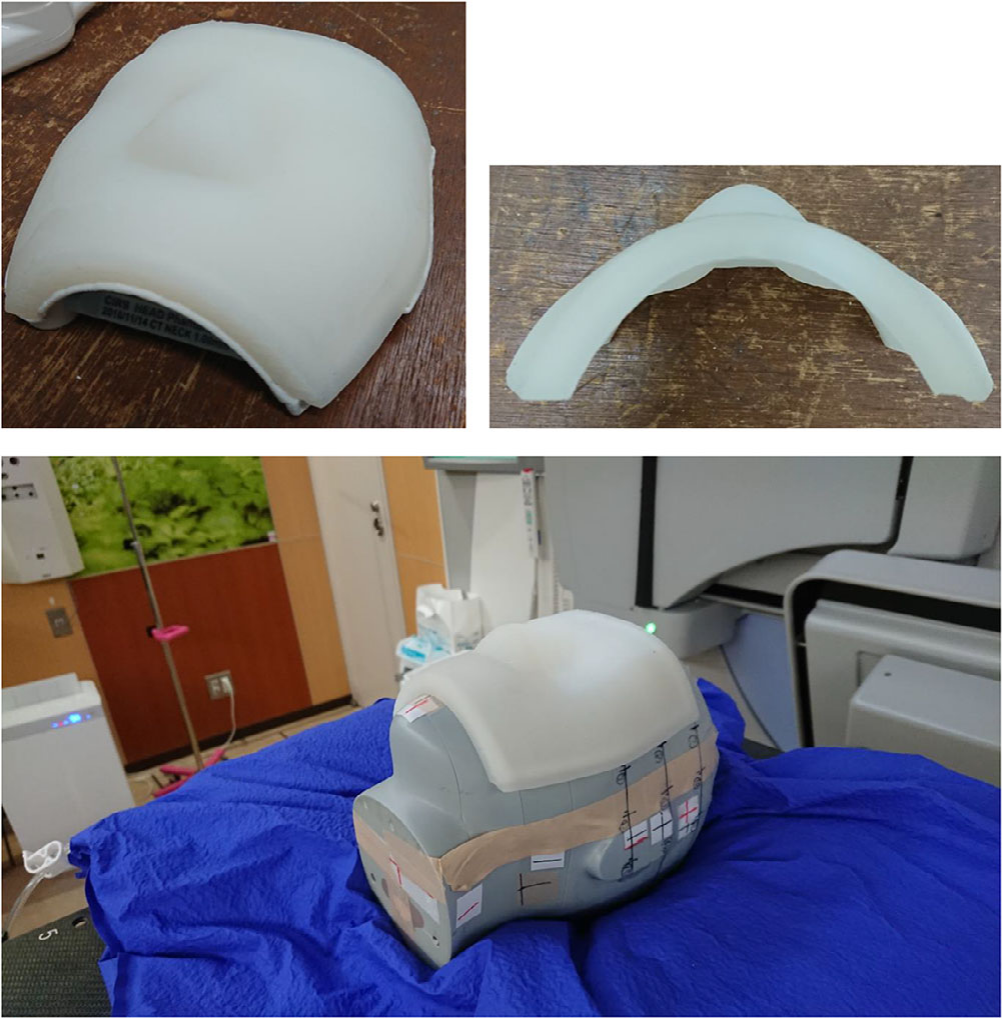
3D printed bolus created for the head phantom and bolus installed on the head phantom. 3D, three‐dimentional.

A 1‐cm‐thick bolus was developed to fit the facial area of the head phantom (CIRS Inc., Norfolk, VA, USA). Based on the CT scan images of the head phantom, a cast was made with gypsum to fit the face using a 3D printer (ZPrinter450, 3D SYSTEMS, SC, USA). The 3D‐printed bolus was formed by pouring silicon rubber material (Ecoflex00‐30, Smooth‐On, Inc., PA, USA) into the created mold and by solidifying it. Figure 2 shows the 3D‐printed bolus.

### 2.5 Skin surface dosimetry with a head phantom

**FIGURE 3:**
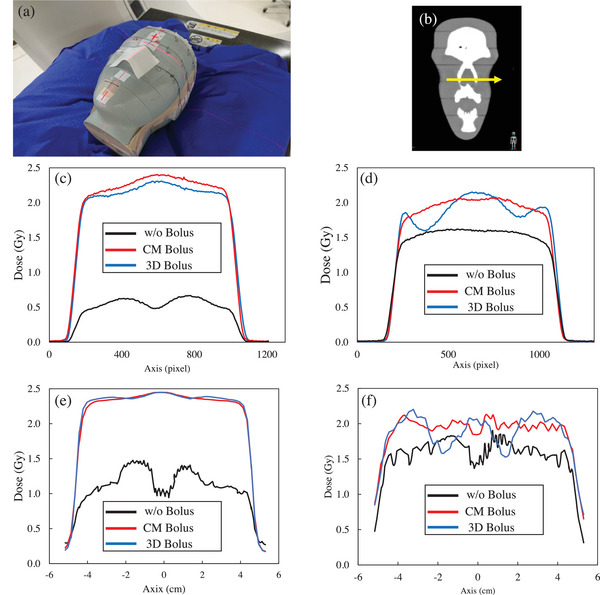
TLD sheet placed on the head phantom and dose profile measured on the TLD sheet. (a) TLD sheet placed on the head phantom, (b) CT image showing the location of the dose profile, (c) dose profile with 4‐MV X‐ray, (d) 6‐MeV electron beam dose profile, (e) TPS dose calculation profiles for 4‐MV X‐ray, and (f) TPS dose calculation profiles for 6‐MeV electron. 3D, three‐dimensional; CM, commercial; CT, computed tomography; TLD, thermoluminescence dosimeter; TPS, treatment planning system.

The CT scan images of the head phantom were acquired without a bolus (w/o bolus), with a 1‐cm‐thick commercial bolus (CM bolus), and with the 3D‐printed bolus (3D bolus). The acquired CT scan images were used to create treatment plans in RayStation. The dose calculation algorithms for treatment planning were CCC for X‐ray therapy and MC for electron beam therapy, and the dose calculation grid size was 2 mm. The dose calculation grid size was equivalent to that of clinical practice. Each treatment plan was created to irradiate 200 MU with an irradiation field of 10 × 10 cm from a gantry angle of 0°. The sources used were 4‐MV X‐ray and 6‐MeV electron beam. A 1‐mm‐thick ROI was created on the nasal surface, which was evaluated as a structure simulating the skin. Surface dosimetry with a TLD sheet was performed using the irregularly shaped nose area of the head phantom. Irradiation was performed under the same conditions as in the treatment plan, and the irradiation device was Elekta Synergy. A 5 × 5‐cm TLD sheet was placed in close contact with the nose of the head phantom during irradiation (Figure 3a). Measurements were taken three times for each of the three bolus conditions. The nasal profile (Figure 3b) of the measured data was calculated using ImageJ.

## 3 RESULT

### 3.1 Association between TLD sheet measurements and irradiation dose

**FIGURE 4:**
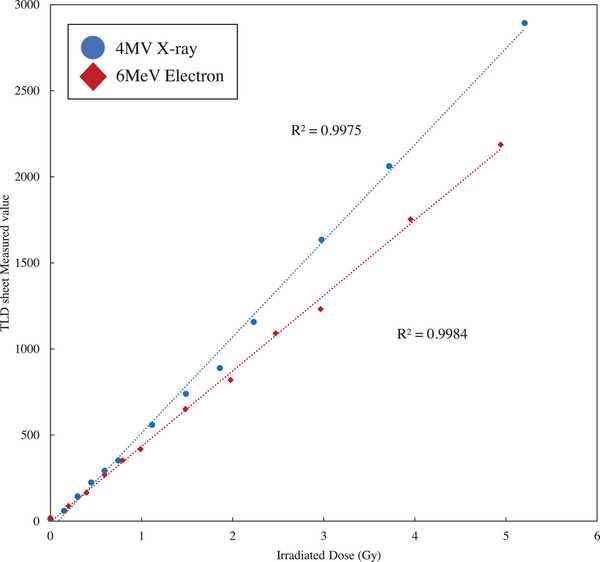
Relationship between irradiation dose and TLD sheet measurement values. TLD, thermoluminescence dosimeter.

Figure 4 shows the association between the irradiation dose and TLD sheet measurements. The *R* ^2^ values for the linear approximation of the measurements were 0.9975 for the 4‐MV X‐ray therapy and 0.9999 for the 6‐MeV electron beam therapy.

### 3.2 Surface dosimetry with TLD sheets and treatment plan values

The results of the surface dose measured by the TLD sheet and the TPS calculated dose are shown in Table 1. In both X‐ray and electron beam, TLD sheet measurements were higher with bolus than without bolus. This trend was also true for the calculated TPS values, and there was no discrepancy between the measured and calculated values. The difference between the TLD sheet measurements and the TPS calculated doses with and without bolus was 0.08 and 0.87 Gy for X‐ray 4‐MV and 0.01 and 0.13 Gy for electron beam 6‐MeV, respectively.

**TABLE 1:** TLD sheet measurements and TPS calculations in flat phantom.

	X‐ray 4‐MV		Electron 6‐MeV	
	with bolus (Gy)	without bolus (Gy)	with bolus (Gy)	without bolus (Gy)
TLD sheet measured dose	1.91 ± 0.06	0.38 ± 0.03	2.01 ± 0.05	1.44 ± 0.05
TPS calculated dose	1.99	1.21	2.00	1.57

Abbreviations: TLD, thermoluminescence dosimeter; TPS, treatment planning system.

### 3.3 Treatment plans in the head phantom

**FIGURE 5:**
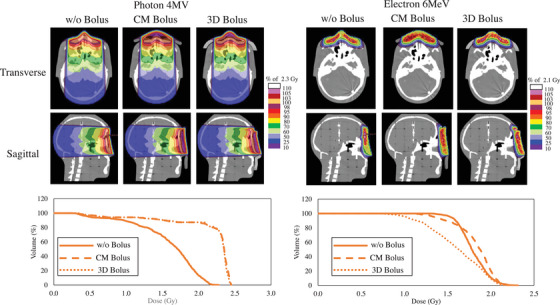
Treatment plan and DVH of skin ROI created near the nose with various bolus placements of w/o bolus, CM bolus, and 3D bolus. 3D, three‐dimensional; CM, commercial; DVH, dose volume histogram; ROI, region of interest; w/o bolus, without a bolus.

Figure 5 shows the dose distributions calculated using TPS for each bolus condition. Higher doses were distributed on the skin surface with the bolus compared with w/o a bolus for both the 4‐MV X‐ray therapy and 6‐MeV electron beam therapy. The dose distribution in the 3D bolus followed the shape of the phantom nose, unlike that in the CM bolus. In the 6‐MeV electron beam therapy, a high‐dose region existed inside the bolus in the 3D‐printed bolus. Figure 5 shows the dose volume histogram (DVH) parameters of the skin. The 4‐MV X‐ray beam presented with an increased skin dose for the CM bolus and 3D bolus compared with w/o a bolus. There was a minimal difference in the DVH curve between the CM bolus and 3D bolus. For the 6‐MeV electron beam therapy, the DVH curve in the 3D bolus was more likely to be lower than that in w/o a bolus and the CM bolus. The CM bolus had higher doses than the w/o a bolus in the high‐dose region.

### 3.4 Surface dosimetry of the head phantom with the TLD sheets

Figure 3(c) and (d) shows the average dose profiles under each bolus condition for the TLD sheets placed on the phantom surface. The 4‐MV X‐ray therapy and 6‐MeV electron beam therapy w/o a bolus had lower doses than those with the CM and 3D bolus. The 4‐MV X‐ray therapy with the CM bolus and that with the 3D bolus were generally comparable in terms of profile shape. However, the CM bolus was more likely to have a higher dose in the high‐dose region. In the 6‐MeV electron beam therapy, the shape of the dose profile differed between the CM bolus and the 3D bolus. Figure 3(e) and (f) shows the dose profiles of the 4‐MV X‐ray and 6‐MeV electron beams, respectively, calculated by TPS under the same conditions as the measurements. For X‐rays, the calculated dose profile was generally similar to the measured dose profile, but the calculated dose was higher than the measured dose. In the case of electron beams, the measured and calculated doses for the profile shape were generally similar, and the dose difference tended to be smaller than that for 4‐MV X‐ray.

## 4 DISCUSSION

This study evaluated the feasibility of the skin surface measurement in radiotherapy using TLD sheets and skin doses in the 3D‐printed bolus. Several papers have been reported on the development of 3D printed boluses.^7^, ^10^, ^12^, ^13^ All of these studies reveal the effectiveness of using 3D printed bolus in reducing the air gap. Regarding air gap, Dyer et al. reported that > 5 mm air gap decreases the accuracy of dose delivery.^14^ They mentioned in this paper that 3D printed bolus has the potential to reduce such a large air gap. The advantage of 3D printed bolus is the reduction of a large air gap due to the simplicity of placement and improved reproducibility, and this advantage was reproduced in the 3D printed bolus developed in this study. This is expected to reduce dose inhomogeneity associated with increased air gap due to bolus placement failure and contribute to increased treatment efficacy.

The results obtained in this study and comparisons with previous studies suggest that the TLD sheet has certain dosimetric capabilities. The TLD sheet is extremely thin and can be fitted and placed in complex geometries such as the nose, ears, and skull, making it a potential tool for direct measurement of skin doses. Due to the ease of fit and the human body equivalence of the TLD material, it is assumed that the accuracy of skin dose prediction will be higher than that of film. It is expected to provide new insights into the prediction of skin toxicity through highly accurate skin dosimetry that reduces the errors of conventional dosimetery with large partial volume effects. In addition, the extremely thin, patient‐fit design facilitates in vivo dosimetry directly on the patient.

The measured values of the TLD sheets with respect to the dose presented with a linear relationship up to 6 Gy. Hence, dosimetry can be performed with the TLD sheets based on the association between the TLD sheet measurements and irradiation doses. The association between the measured values and the irradiation doses differs based on the energy. For the TLD elements, there were differences in terms of response for each energy,^15^, ^16^ and the TLD sheets had similar characteristics. Kato et al. evaluated the possibility of proton dosimetry using the TLD sheets and reported that the dose was associated with the TLD sheet measurements.^11^ Therefore, the association between the measured value and the irradiation dose at each energy using the TLD sheets for dosimetry should be calculated.

In X‐ray, the difference between the treatment plan and measured values at the beam injection surface in the w/o bolus was 68.6%, indicating that the calculated value in the TPS was higher. In contrast, the error was reduced to 4.0% with the bolus in place. The depth of the buildup area for the 4‐MV X‐ray therapy is approximately 1.5 cm, which is considered a large error between the measured value and the TPS calculation value caused by the insufficient charged particle equilibrium at the depth where the TLD sheet was set in the surface dose measurement. This can be attributed to errors in the surface dose calculation in the dose calculation algorithm. According to several reports, the CCC used in this study had calculation errors in surface dose. For example, Farhood et al. reported that the errors in the CCC dose calculation for a 10 × 10‐cm irradiation field were −10.3% at < 2 mm and 4.99% at < 10 mm from the X‐ray incident surface.^17^ Cao et al. reported an error of 11.37 % for skin doses at CCC.^18^ In addition, Chung et al. showed that the dose calculations of commercial treatment planning devices may overestimate surface doses by >10% relative to measured doses.^19^ Compared with their study, the difference between the TLD sheet measurements of the surface dose and the treatment plan calculations in this study was large. The TLD sheets used in this study were extremely thin (thickness: 0.2 mm). The treatment planner's calculation results were based on the average dose in ROIs that resemble the TLD sheets, which are thicker than the actual TLD sheets because of the pixel size. This might have caused the larger error between the measured and calculated values in this study. Further, Chung et al. reported dose errors of > 10% at the very surface. For the local evaluation of the very surface, there might be large errors at > 15% between the TPS and the measurements.^19^ The error between the measured values and the TPS calculation values in electron beam was smaller than that in X‐ray. In particular, the magnitudes of error were 8.4% without bolus and 0.1% with bolus. This could be caused by the use of MC in the calculation algorithm for electron beam. Electron MC calculations of commercially available TPSs had a good agreement with actual measurements.^20^, ^21^ In addition, Cao et al. reported that in the buildup region, the MC calculation results were closest to the measured values among the various dose calculation algorithms.^18^ The error between the treatment planning calculations and the TLD sheet measurements in this study could be attributed to the difference between the thickness of the TLD sheet and the thickness of the contour evaluated by the TPS, as in the case of X‐ray.

Based on the dose distribution in the head phantom by TPS and DVH with the 4‐MV X‐ray therapy, the increase in dose in the skin ROI by the 3D bolus was smaller than that by the CM bolus. Kong et al. revealed that the 3D‐printed bolus can provide an improved dose to targets near the skin surface compared with commercially available boluses.^6^ Fujimoto et al. similarly reported an improved coverage of tumors near the skin with the 3D‐printed bolus compared with the commercially available bolus.^9^ In contrast, in our study, the doses to the skin in the 3D bolus and CM bolus were almost similar in the 4‐MV X‐ray therapy. This might be attributed to differences in the ROIs used for evaluation and treatment plans. The ROIs evaluated in this study were those that simulated the skin localized in the vicinity of the skin. In contrast, their study evaluated ROIs with some thickness in the depth direction. Notably, their study showed no significant difference between the 3D‐printed bolus and commercially available bolus for high doses near *D*_max_. Therefore, the difference according to the bolus was more likely to occur in the area where the dose begins to decrease at some distance from the skin in the deep direction. Moreover, there was no significant difference between the CM bolus and the 3D bolus in the dose just below the skin. In addition, in this study, the irradiation was performed with a single field with a gantry angle of 0°, and the MU was similar in all bolus situations. In contrast, Kong et al. and Fijimoto et al. evaluated the treatment plans with IMRT and oblique beam, respectively. Further, the MU was adjusted to deliver a specific amount of dose to the tumor. Therefore, the near‐skin dose in the 3D bolus created in this study can be increased compared with that in the CM bolus by optimizing the treatment plan.

In the TLD sheet measurements, the dose increase effect with and w/o bolus was observed at 4‐MV and 6‐MeV. This is similar to the results of a previous study using a different measurement. Further, the current study has proven this notion using a thinner and more convex dosimetry device.^3^ The use of the TLD sheets that follow the uneven geometry facilitates a better measurement of the actual dose of the skin than before, and such results have not been reported by previous studies. The surface dosimetry results indicate that the skin dose was overestimated in the dosimetry of the w/o bolus treatment plan. In X‐rays, the surface dose evaluation resulted in a significant overestimation of the TPS calculated values compared with the TLD sheet measurements. This was also the case for the dose profile results calculated by the TPS (Figure 2e and f). For electron beams, the dose difference between TPS calculated and measured profiles was small for X‐rays. This can be attributed mainly to differences due to the TPS calculation algorithm. Thus, previous evaluations of treatment plans have included errors in dose calculation algorithms. However, the TLD sheets in this study could evaluate surface doses by measurement.

Compared to the CM bolus, the dose distribution with the 3D bolus showed that the high dose spread was distributed along the skin geometry. The dose in the high‐dose region was higher in the CM bolus (Figure 3c). The dose distribution of the TPS shown in Figure 5 indicates that the 103% isodose line was distributed near the area where the TLD sheet was placed in both the CM bolus and 3D bolus. This may be useful when there is a need to distribute the high dose range over a wider skin area. In contrast to this, in X‐rays, the dose profile shapes of the CM bolus and 3D bolus were almost similar. This is also expected from the fact that the dose values of the skin ROI of the CM bolus and 3D bolus were almost similar in the DVH curve. The 3D bolus is made of silicone and may have had a slightly reduced dose relative to the CM bolus of water‐equivalent material. According to previous studies, the 3D‐printed bolus resulted in a limited increase in skin surface dose, and this result is also consistent with these trends. In the electron beam, there was a difference in the profile shape between the CM bolus and 3D bolus (Figure 3d). The CM bolus presented with a mountainous dose profile. Meanwhile, the 3D bolus presented with a dose profile with peaks and valleys along the shape of the nose area. The dose distribution shown in Figure 5 confirmed that the 3D bolus followed the shape of the body surface, and the dose absorption also occurred within the bolus, which caused the unevenness in the dose profile near the skin. As mentioned in the preceding text, the skin dose can be increased by optimizing the treatment plan considering the 3D bolus.

**FIGURE 6:**
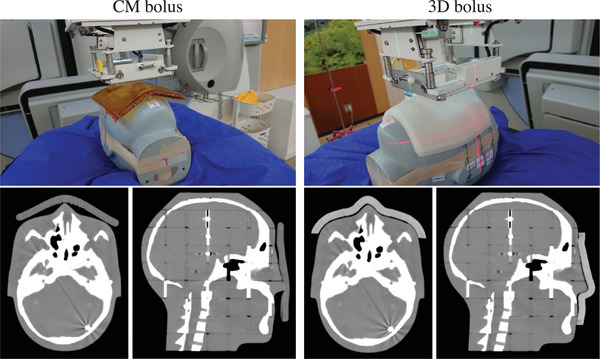
Geometry of measurement experiments performed with CM and 3D boluses placed in the phantom and CT images acquired with each bolus placed in the head phantom. 3D, three‐dimensional; CM, commercial; CT, computed tomography.

In this study, a 3D bolus was created that fits the shape of the head phantom. Phantom and 3D bolus CT scan images presented with a small gap between the bolus and skin surface relative to the CM bolus (Figure 6). Dipasquale et al. reported that the 3D printed bolus reduced the air gap between the bolus and the body surface.^22^ Similarly, Robar et al. showed an improved adhesion between the body surface and the 3D‐printed bolus.^7^ Reducing the air gap between the bolus and the skin surface is beneficial. That is, it reproduces bolus placement. Thus, it can contribute to a more accurate treatment by improving the reproducibility of treatment plans. This is considered more important in high‐precision radiotherapy such as IMRT and stereotactic radiotherapy.

This study had a limitation. That is, only one irradiation condition for the fixed MU with a gantry angle of 0° was applied to the head phantom. The reason for performing the measurements under these conditions was to eliminate as much uncertainty as possible dependent on the treatment plan and to correctly assess the previously unidentified capacity of the TLD sheets. In the past, angled beams have been reported to cause significant differences between measurements and dose calculations,^17^, ^18^ and there was concern that these factors would obscure the results of this study. A simple beam setup was used to characterize the buildup area in X‐rays. Understanding these physical characteristics will be helpful in understanding the characteristics of dosimeters and their adaptation to clinical use. In electron beams, we believe that even the beam configuration used in this study is generally sufficient for clinical situations. In actual treatments, the beam angle and MU values are adjusted according to tumor localization, shape of the irregularities, and bolus material. Therefore, the benefits of the dose distribution formed during treatment might have differed in this study. In addition, optimized treatment plans such as IMRT and ablative stereotactic radiotherapy, as well as measured experiments with multi‐field irradiation, may further clarify the clinical benefits of 3D‐printed bolus.

## 5 CONCLUSION

This study evaluated the feasibility of surface dosimetry using TLD sheets and 3D‐printed boluses. Since the energy dependence of the TLD sheet has been confirmed, the TLD sheet can be used for an accurate dosimetry by identifying an appropriate association between the dose and the measured value for each energy. Errors between TLD sheets and dose calculations were observed. Thus, they may represent errors in surface dose assessments conducted using the dose calculation algorithm. This result indicates that highly accurate skin surface measurements can be achieved even on skin surfaces where errors occur in dose calculations, thereby ignoring the partial volume effect of the detector, the TLD sheet. The use of the 3D‐printed bolus did not increase skin doses under the conditions in this study. However, it improved the fit on the body surface, which is effective in improving reproducibility.

## AUTHOR CONTRIBUTIONS

*Writing the manuscript*: Yuya Miyasaka. *Dose measurement*: Yuya Miyasaka, Yoshifumi Yamazawa, and Hongbo Cahi. *Data analysis*: Yuya Miyasaka and Hikaru Souda. *Conceptualization*: Yuya Miyasaka and Mayumi Ichikawa. *Methodology*: Yuya Miyasaka, Mayumi Ichikawa and Akira Takagi. *Creating 3D printed bolus*: Takagi Akira. *Review manuscript*: Hikaru Souda, Miyu Ishizawa, and Hiraku Sato. *Creating plan*: Yuya Miyasaka. *Clinical integration*: Mayumi Ichikawa and Hiraku Sato. *Clinical review*: Hiraku Sato. *Management and coordination responsibility for the research activity planning and execution*: Takeo Iwai.

## CONFLICT OF INTEREST STATEMENT

The authors declare no conflicts of interest.

## Data Availability

Research data are stored in an institutional repository and will be shared upon request to the corresponding author.
